# A multi-plex protein expression system for production of complex enzyme formulations in *Trichoderma reesei*

**DOI:** 10.1093/jimb/kuac027

**Published:** 2022-12-13

**Authors:** Venkataramanan Subramanian, Samuel J Farmer, Kelsey L Heiland, Kyle T Moore, Todd A Vander Wall, Weiman Sun, Yogesh B Chaudhari, Michael E Himmel, Stephen R Decker

**Affiliations:** Biosciences Center, National Renewable Energy Laboratory, 15013 Denver West Parkway, Golden, CO 80401, USA; Biosciences Center, National Renewable Energy Laboratory, 15013 Denver West Parkway, Golden, CO 80401, USA; Biosciences Center, National Renewable Energy Laboratory, 15013 Denver West Parkway, Golden, CO 80401, USA; Biosciences Center, National Renewable Energy Laboratory, 15013 Denver West Parkway, Golden, CO 80401, USA; Biosciences Center, National Renewable Energy Laboratory, 15013 Denver West Parkway, Golden, CO 80401, USA; Biosciences Center, National Renewable Energy Laboratory, 15013 Denver West Parkway, Golden, CO 80401, USA; Biosciences Center, National Renewable Energy Laboratory, 15013 Denver West Parkway, Golden, CO 80401, USA; Biodiversity and Ecosystem Research, Institute of Advanced Study in Science and Technology (IASST), Guwahati 781035, India; Biosciences Center, National Renewable Energy Laboratory, 15013 Denver West Parkway, Golden, CO 80401, USA; Biosciences Center, National Renewable Energy Laboratory, 15013 Denver West Parkway, Golden, CO 80401, USA

**Keywords:** *Trichoderma reesei*, Foot-and-mouth-disease virus 2A-peptide, Cellobiohydrolase, Endoglucanase, β-D-glucosidase, Protein expression, Cellulase activity, AZCL-HE-cellulose

## Abstract

Heterologous protein production has been challenging in the hyper-cellulolytic fungus, *Trichoderma reesei* as the species is known for poor transformation efficiency, low homologous recombination frequency, and marginal screening systems for the identification of successful transformants. We have applied the 2A-peptide multi-gene expression system to co-express four proteins, which include three cellulases: a cellobiohydrolase (CBH1), an endoglucanase (EG1), and a β-D-glucosidase (BGL1), as well as the enhanced green fluorescent protein (eGFP) marker protein. We designed a new chassis vector, pTrEno-4X-2A, for this work. Expression of these cellulase enzymes was confirmed by real-time quantitative reverse transcription PCR and immunoblot analysis. The activity of each cellulase was assessed using chromogenic substrates, which confirmed the functionality of the enzymes. Expression and activity of these enzymes were proportional to the level of eGFP fluorescence, thereby validating the reliability of this screening technique. An 18-fold differencein protein expression was observed between the first and third genes within the 2A-peptide construct. The availability of this new multi-gene expression and screening tool is expected to greatly impact multi-enzyme applications, such as the production of complex commercial enzyme formulations and metabolic pathway enzymes, especially those destined for cell-free applications.

## Introduction

The ascomycete fungus, *Trichoderma reesei*, is primarily studied for its lignocellulose degradation abilities. The long-standing interest in this fungus is primarily due to its ability to produce high titers of cellulase and xylanase enzymes. Up to 100 g per liter of protein is known to be produced by *T. reesei* in industrial settings (Cherry & Fidantsef, 2003). In contrast, heterologous protein production has been a challenge using this organism. This history arises from the limited understanding of regulation, expression, and modulation of protein production and secretory mechanisms for this fungus. Furthermore, post-translational mechanisms also play a major factor in the expression of such proteins. Efforts to express non-cellulolytic enzymes have yielded only limited success (Peterson & Nevalainen, 2012). Extensive research has been undertaken to improve protein synthesis in *T. reesei* via the development of advanced promoters, which include constitutive, tunable, and the recently developed synthetic promoters (Fitz et al., 2018; Li et al., 2012; Rantasalo et al., 2019). Protein secretion has also been investigated, leading to our current understanding of the pathways involved in this process (de Paula et al., 2019; Pakula et al., 2005; Saloheimo & Pakula, 2012; Stappler et al., 2017).

There are specific challenges associated with expressing heterologous proteins; these include efficiency of DNA transformation, site-directed integration targeting, and screening/identification of positive protein-expressing transformants. Whereas two of these challenges have been addressed as a consequence of the development of improved transformation techniques (de Groot et al., 1998; Hazell et al., 2000; Te'o et al., 2002; Zhong et al., 2007), the development of non-homologous end-joining mutants (Guangtao et al., 2010; Jorgensen et al., 2014), and CRISPR tools (Fonseca et al., 2020; Liu et al., 2015), the screening of protein-expressing transformants still remains a hurdle, especially when dealing with difficult-to-express and larger heterologous proteins. Our laboratory has reported an efficient protein expression and screening technique using the foot-and-mouth disease virus 2A-peptide (Subramanian et al., 2017). This approach joins two proteins with a 22-amino acid 2A-peptide that auto-catalyzes the separation of the two expressed proteins during translation. Whereas these two proteins and their connecting 2A-peptide sequence are encoded by a single mRNA transcript, they are auto-separated due to the “ribosomal-skip” mechanism deployed during the translation process (Donnelly et al., 2001a; Donnelly et al., 2001b; Radcliffe & Mitrophanous, 2004).

**Fig. 1.**
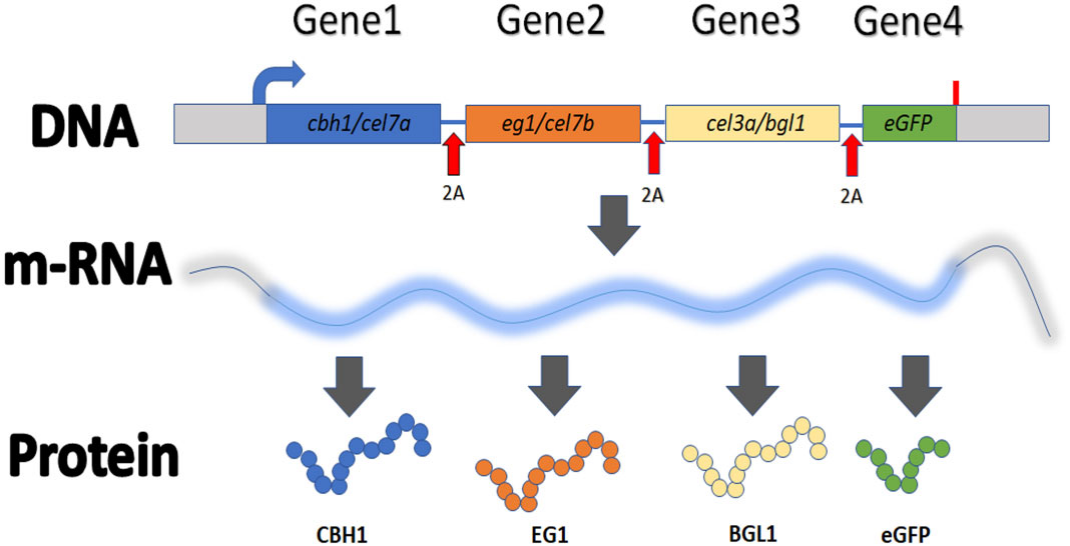
Schematic representation of the multi-cistronic protein expression approach. Four genes, namely, *cel7a, cel7b, bgl1*, and *eGFP* are separated by 2A-peptide variants in the plasmid construct. Upon transcription, a single mRNA transcript is generated, which is translated into four independent proteins: CBH1, EG1, BGL1, and eGFP. Auto-separation occurs at the 2A-peptide residues (shown as red arrows). Blue, curved arrow represents the initiation of translation. Stop codon is represented by red line after *eGFP.*

Considering the natural ability of this fungus to produce cellulolytic enzymes, it would be advantageous, as well as prudent, to exploit this fungus for the production of customized ligno-cellulolytic enzyme cocktails. Here, we have implemented the 2A-peptide approach for this application using the newly developed “plug-and-play” plasmid expression vector that acts as a chassis for expressing up to five genes simultaneously under a single promoter. There is presently no known protein multiplexing tool available in this fungus. Here, we demonstrate functional expression of a complete minimal cellulase cocktail, which includes a cellobiohydrolase (CBH1, CEL7A), an endoglucanase (EG1, CEL7B), and a β-D-glucosidase (BGL1, CEL3A). As cellulases make poor selection markers, the expression of the cellulase enzymes is monitored using enhanced green fluorescent protein (eGFP) fluorescence as an indicator. Because all four proteins originate from a single transcript, the upstream cellulases are expressed prior to the terminal eGFP (Fig. 1). eGFP expression is thus a strong indicator of upstream protein processing. We have further evaluated and quantified the expression levels of each of the expressed genes within the 2A-peptide construct using quantitative reverse transcription polymerase chain reaction (PCR) and immunoblot analysis.

## Materials and Methods

### Strains and Growth Conditions

The *T. reesei* strains SV004, AST1116, and JLT102A, all of which are derivatives of QM6a, were used in this study. They were routinely maintained on potato dextrose agar (PDA) medium. Transformants were selected on PDA containing hygromycin (100 µg/mL) and 1% Triton X-100 (PDHX) medium, where Triton X-100 served as the colony restrictor. For the screening of transformants, strains were grown in Mandels Andreotti (MA) minimal medium containing 5% glucose (MAG). Transformants were selected on MAG + H medium. AST1116 is a *cel7a*-deleted derivative of strain QM6a. The SV004 strain is a transformant of AST1116 containing the *cel7a*-2A-eGFP construct (Subramanian et al., 2017). JLT102A is a recombinant strain of AST1116 that contains the native *cel7a* gene under the control of the *eno* promoter (Linger et al. 2015).

### Plasmid Construction

**Fig. 2.**
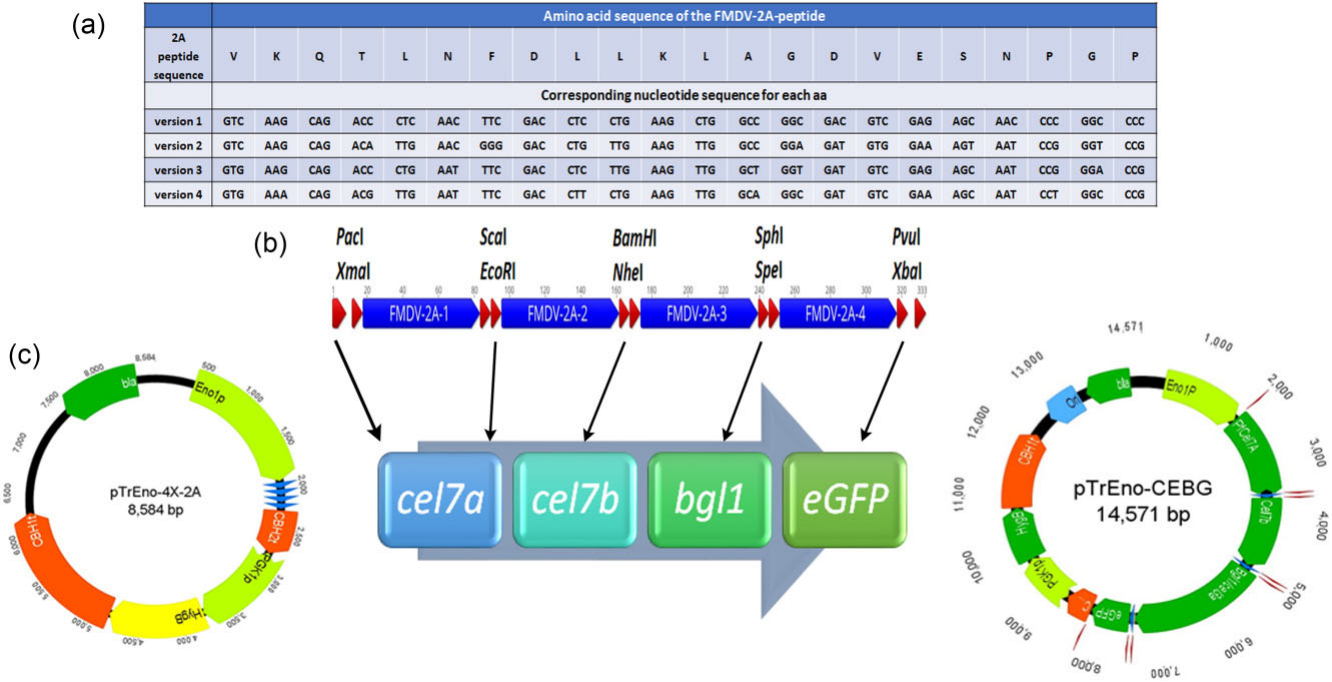
Construct design for expression of the cellulase enzyme cocktail. **a.** Amino acid sequence and the four nucleotide variants of the FMDV-2A peptide sequence. **b.** The 4X-2A cloning cassette containing the different restriction sites is shown. Blue bars indicate 2A-peptide sequences and red arrows represent the different restriction sites. **c.** Insertion of the four genes into the pTrEno-4X-2A chassis vector to yield the pTrEno-CEBG vector. Black arrows above the individual genes indicate the site of insertion within the gene cassette.

The base vector pTrEno-4X-2A containing the cloning sites was constructed as follows: The four 2A-peptide-sequences-containing g-block was obtained from IDT Technologies. This g-block specifically contained an 83 bp *eno1* promoter sequence followed by four 2A-peptide sequences in tandem separated by restriction sites (Fig. 2). This was followed by 84 bases of the *cbh2* terminator sequence. A *Pac*I site was included immediately after the eno1 promoter sequence, while an *Xba*I site was included immediately after the stop codon at the 3′ end of this 2A-peptide cassette. A schematic representation of this cloning vector is shown in Fig. 2. This g-block fragment was cloned into the temporary vector pJET1.2 to obtain pJET-2A-ver3 plasmid. This pJET1.2 clone containing the 500 bp sequence was restricted with *Pac*I and *Xba*I enzymes and inserted into the pTrEno-PF plasmid (Linger et al., 2015) at the *Pac*I and *Xba*I sites to obtain the pTrEno-4X-2A plasmid. Plasmid sequence was confirmed by DNA sequencing.

The first gene, *Pfcel7a*, from *Penicillium funiculosum*, was amplified from the pTrEno-PF vector using the primers SV-33 and SV168, such that *Pac*I and *EcoR*I sites were inserted into the 5′ and 3′ ends of the amplified product, respectively (Taylor et al., 2018). A list of primers used in this work is provided in Supplementary Table 1. The second gene, *cel7b* (Protein ID 122081), was amplified from a plasmid containing the cDNA (Genscript, Inc., Piscataway, NJ, USA) using the primers SV-230 and SV-231 such that *BamH*I and *Nhe*I sites were introduced at the 5′ and 3′ ends, respectively, of the amplified product. The third gene, *bgl1*/*cel3a* (Protein ID 76672), was amplified from genomic DNA using the primers SV-346 and SV-347 so that *Spe*I and *Sph*I restriction sites were introduced at the 5′ and 3′ ends of the PCR product, respectively. The reverse primer also introduced a 6X-histidine tag to allow detection of protein expression. Each of the three genes was introduced sequentially into the pTrEno-4X-2A vector using restriction cloning at the respective restriction sites to give the plasmid pTrEno-Cel7A-Cel7B-Bgl1. Note that this plasmid had lost the first FMDV-2A peptide sequence. The *eGFP* gene was introduced into this construct using the following steps: The eGFP gene was amplified from the pTrEno-C2G vector (Subramanian et al., 2017) using the primers SV-246 and SV-79. The pTrEno-Cel7A-Cel7B-Bgl1 plasmid was amplified using the primers SV-75 and SV-278, followed by *Dpn*I treating this PCR product. Both fragments were then ligated using an infusion cloning protocol (Takara Bio USA, Inc., Mountain View, CA, USA). The final plasmid, pTrEno-Cel7A-Cel7B-Bgl1-eGFP (pTrEno-CEBG), that was generated is shown in Fig. 2. The confirmation of DNA sequence was done by sequencing. It should be noted that whereas the first three genes lacked the stop codon, the last gene, *eGFP*, contained the stop codon, thereby allowing continuous translation of all four proteins.

### Transformation of Strains

Spore preparation of the AST1116 strain was carried out per the protocol described in Linger et al. (2015). Briefly, spores were harvested from three day-grown PD plates, followed by washing and resuspending in 10% sorbitol prior to freezing at –80°C. Plasmids were linearized with *Sbf*I enzyme followed by purification using the DNA Clean and Concentrator-5 kit (Zymo Research Corp., Irvine, CA, USA). One microgram of this linearized plasmid was electroporated using a Bio-Rad Gene Pulser (Bio-Rad Laboratories, Inc., Hercules, CA, USA) using the following conditions: 1.8 kV, 25 µF, and 800 Ω. This was followed by the incubation of the spore suspension on ice for a few minutes. One milliliter of complete medium containing lactose (CML) was added to this electroporation mixture and transferred to a six-well tissue culture plate for overnight incubation at room temperature. PDHX plates were then spread with ∼200 µL of this electroporation mixture and incubated at 30°C for three days in an illuminated incubator to allow colony development.

### Screening of Transformants for Gene Expression

Transformant colonies growing on PDHX plates were individually picked and transferred to 24-well plates containing 2 mL of MAG + H medium and incubated for three days at 30°C for mycelial development. These plates were directly visualized using FluorChem Q imaging system (Cell Biosciences, Santa Clara, CA, USA). Quantification of fluorescence was carried out using the FLUOstar Omega plate reader (BMG Labtech GmbH, Germany). Each 24-well plate included the eGFP-expressing strain, SV004, and the eGFP-lacking strain, AST1116, as controls.

### Genomic Confirmation of Transformants and Gene Copy Number Analysis

Genomic DNA was extracted from the transformants using Quick-DNA Fungal/Bacterial Miniprep kit (Zymo Research Inc., Irvine, CA, USA). PCR analysis was carried out using Taq DNA polymerase kit (Qiagen Inc., Germantown, MD, USA). PCR primers SV-82 and JL-383 were used to amplify entire gene cassettes (Supplementary Table 1). Twenty-five nanograms of DNA was used for the PCR reaction. For gene copy number analysis, the Powertrack SYBR Green Master Mix kit was used (ThermoFisher Scientific, Waltham, MA, USA). A standard curve was generated for two representative genes (*cel7b* and *cel3a*) from the CEBG cassette and one single-copy gene *act* (Protein ID 44504) that is natively present in *T. reesei*. Primers used for the PCR analysis are shown in Supplementary Table 1. The copy numbers of *cel7b* and *cel3a* genes were measured using the relative comparison approach with actin as the control gene.

### Invitro Cellulase Activity Assays

The activities of extracellular CBH1, EG1, and BGL1 were measured using 4-nitrophenyl β-D-lactopyranoside (*p*NP-L), 4-nitrophenyl β-D-cellobioside (*p*NP-C), and 4-nitrophenyl β-D-glucopyranoside (*p*NP-G) (Sigma-Aldrich Corp., St. Louis, MO, USA) as substrates, respectively. Briefly, 25 µL of extracellular cell-free broth was added to 96-well plates containing 125 µL of 2 mM *p*NP substrates and incubated at 45°C for 1 hr. The CBH1 expressing strain (JLT102A) and the CBH1-null strain AST1116 were used as controls. Enzyme reactions were stopped by the addition of 25 µL of 1 M sodium carbonate solution and to allow color development. Activity was measured as µmoles of *p*NP released against a calibration curve generated with a standard *p*NP solution (Sigma-Aldrich Corp., St. Louis, MO, USA) at 405 nm.

### Immunoblot Analysis

Transformants grown on 24-well plates were used for immunoblotting analysis as follows: For extracellular protein analysis, cell-free broth was mixed with one-third volume of SDS-PAGE loading buffer and subjected to boiling for 10 min. For intracellular protein extraction, a small mycelial fragment was suspended in 150 µL water and subjected to bead beating for 5 min in a TissueLyserII instrument (Qiagen Inc., Germantown, MD, USA). The protein extract was clarified by centrifugation and transferred to a fresh microcentrifuge tube containing NuPAGE LDS Sample buffer (ThermoFisher Scientific, Grand Island, NY, USA) and boiled for 10 min. Proteins were separated on 4–12% Bolt Bis-Tris PAGE gels in MOPS buffer and transferred to polyvinylidene difluoride (PVDF) or nitrocellulose membranes using a Power Blotter (Thermo Fisher Scientific, Inc., Grand Island, NY, USA) following the manufacturer's instructions. For the detection of CBH1 and EG1, immunoblots were hybridized using custom-raised protein-specific primary antibodies, raised in rabbit (polyclonal) and mouse (monoclonal), respectively, at a dilution of 1:3000. The BGL1 and eGFP immunoblots were hybridized with mouse anti-His and mouse anti-GFP antibodies, respectively (both purchased from Thermo Fisher Scientific Inc., Grand Island, NY, USA), at dilutions of 1:4000 and 1:2000, respectively. Colorimetric detection of CBH1 was carried out using alkaline phosphatase (AP)—conjugated goat anti-rabbit secondary antibody. Chemiluminescence detection of EG1, BGL1, and eGFP was carried out using an horse radish peroxidase (HRP)-conjugated anti-mouse secondary antibody. Both the AP- and HRP-conjugated antibodies were obtained from Thermo Fisher Scientific Inc. (Grand Island, NY, USA).

For quantitative band analysis of the expressed CEBG proteins, serial dilutions of purified proteins were used as standards over the range of 1.2–0.15 µg per loading. Cell-free broth at protein amounts of 25–1.56 µg was used to quantify the concentrations of expressed CEL7A, EG1, and BGL1 proteins. The cell-free broth was obtained from an 8 L fermentation culture, followed by harvesting, vacuum filtration, and concentration of the proteins (Brunecky et al., 2020). Total protein concentration was determined by bicinchoninic acid (BCA) analysis. Total proteins from the cell-free broth were separated on SDS-PAGE, along with the corresponding standard proteins on the same gel. Standard curves were generated for purified CBH1 (from *P. funiculosum)*, EG1 (from *Trichoderma longibrachiatum*, Megazyme Ltd., Bray, Ireland), and an in-house his-tagged CBH1 protein that served as controls for CBH1, EG1, and BGL1, respectively. Protein bands were then analyzed using the FluorChem Q imaging station band analysis software.

### Qualitative Cellulase Activity Assay

Proteins from the three transformants, #28, #47, and #48, which showed expression of all four proteins, were selected for activity testing using the chromogenic cellulose substrate AZCL-HE-cellulose (Megazyme Inc., Chicago, IL, USA). Cellulase activity using this substrate was measured by the amount of soluble blue dye released due to the breakdown of the colored substrate. Briefly, AZCL-HE-cellulose was added to MA medium at a concentration of 200 mg/L. An agar plug containing the mycelial mat was placed on the center of the AZCL-HE-cellulose plate and incubated at 30°C in a lighted incubator and monitored for the growth of the fungus along with the release of the blue dye. Controls used in this analysis were the *cel7a*-deleted strain AST1116, the wild-type strain QM6a, and the cellulase hyperproducing strain Rut-C30.

### RNA Extraction and Real-Time Quantitative Reverse-Transcription PCR Analysis

Fungal cultures were grown in MAG + H media for three days prior to harvesting by vacuum filtration. Mycelia were washed with cold sterile water during the filtration process to remove any media components and then transferred to clean petri plates for snap freezing with liquid nitrogen. Frozen mycelia were transferred to a mortar containing liquid nitrogen, followed by homogenization with a pestle to obtain a fine powder. Approximately 100 mg of the mycelial mass was transferred to RNase-DNase-free tube to which 1 mL of TRI reagent (Molecular Research Center, Cincinnati, OH, USA) was added to resuspend the mycelial mass. RNA extraction was carried out using the manufacturer's protocol and quantified using a NanoDrop spectrophotometer. Twenty micrograms of RNA was then subjected to DNase treatment by mixing 4U of DNaseI (ThermoFisher Scientific, Waltham, MA, USA) in the presence of 1X DNaseI buffer and incubating for 30 min at 37°C. The DNaseI was then inactivated by heating the mixture to 75°C for 10 min. This was followed by further purification using the RNeasy mini-prep protocol (Qiagen Inc., Germantown, MD, USA). Quantification of RNA was carried out using a NanoDrop spectrophotometer.

Five hundred nanograms of RNA was then subjected to reverse transcription using the Superscript IV First-Strand Synthesis System (ThermoFisher Scientific, Grand Island, NY, USA) using the manufacturer's protocol. Quantitative PCR was then carried out in biological triplicates using the Powertrack SYBR green master mix kit (ThermoFisher Scientific, Grand Island, NY, USA). A total of 2 µL of the reverse transcription mixture was used as the template. Gene specific primers for *cel7A, cel7B, bgl1* and *eGFP* were used for quantification (Supplemental Table 1). The actin gene was used as the internal control for normalization purposes. The PCR consisted of the following parameters: 95°C for 2 min, 95°C for 5 s, and 60°C for 30 s. RNA quantification was done using the ΔΔCt approach. Briefly, the Ct values obtained for each of the biological replicates were normalized to the actin values to obtain the Δct values. This was followed by subtracting the ΔCt values of the transformants with that of the untransformed control strain QM6a to obtain the ΔΔCt values. Change (x-fold) was then calculated using the 2^^^Ct formula.

## Results and Discussion

We have previously shown that two proteins can be expressed simultaneously using the 2A-peptide approach (Subramanian et al., 2017). Although this approach is applicable for screening expression of one protein, a multiple protein expression platform for this fungus is yet to be developed. To develop this fungus as a protein/metabolic pathway compatible expression host, either a suite of resistance markers for transformation of multiple plasmids or a multi-cistronic protein-expressing strategy must be developed. Undoubtedly, there are only a few selection markers available for this fungus. Thus, the second approach is more relevant for hosts such as *T. reesei*. Here, we report the first example of successful multiplexed protein expression in this fungus with our goal being application to the production of commercial enzyme cocktails, as well as multiple enzymes for cell-free production of bioproducts.

### Multi-Cistronic Protein Expression Approach

Our previous work revealed that two genes can be functionally co-expressed in *T. reesei* and their expression could be monitored by eGFP fluorescence (Subramanian et al., 2017). Here, we tested the expression of a simple cellulase enzyme cocktail consisting of a CBH1, EG1, and BGL1. To obtain a functional level of expression, each gene must be separated by a 2A-peptide sequence to achieve discrete functional proteins upon translation. The overall molecular process involves the transcription of a single mRNA molecule that encodes the four different proteins, all separated by 2A-peptides. Upon translation, the four proteins will be separated into independent peptides by the “ribosomal-skip” mechanism, wherein the peptidyl (2A)-tRNA^Gly^ ester linkage to the glycyl-tRNA in the ribosomal P-site will be hydrolyzed, thereby releasing the polypeptide from the ribosomal translational complex. A schematic representation of the multi-cistronic cellulase-cocktail expression approach is shown in Fig. 1. It is worth mentioning that no stop codons were included after any gene, except the last gene *eGFP*. Thus, translation termination is expected to occur only after the fourth gene, *eGFP*, is translated.

### Development of 2A-Peptide Expressing Chassis Vector Using Restriction-Based Cloning

To obtain a four-gene containing expression vector, we generated a chassis plasmid containing four FMDV 2A-peptide variants separated by gene insertion sites to allow cloning of multiple genes into this vector. We used four different codon variants of the 22 amino acid peptide, which are shown in Fig. 2a. This step was intended to avoid repeat sequences being introduced into the genome to prevent recombination events and subsequent looping-out of the genes. Furthermore, we also designed unique restriction sites into the chassis vector lying between the 2A-peptide sequences. These sites included the *Xma*I site before variant 1, the *Sca*I and *EcoR*I sites between variants 1 and 2, the *BamH*I and *Nhe*I sites between variants 2 and 3, the *Sph*I and *Spe*I sites between variants 3 and 4, and the *Pvu*I sites after variant 4 (Fig. 2b). These restriction sites are in addition to the already existing *Pac*I and *Xba*I sites immediately after the promoter and before the terminator sequences, respectively. This chassis vector was used to introduce the four genes, *cel7a, cel7b, cel3a*, and *eGFP*, resulting in the generation of the pTrEno-CEBG vector (Fig. 2c). Although, in theory, five genes can be cloned into this vector using the available restriction sites. It should be noted that *eGFP* was introduced as the last gene in that order to use it as a fluorescent marker for detecting protein expression, based on its best functionality at this position (Subramanian et al., 2017). Two of the genes, *cel7a* and *eGFP*, were heterologous proteins, originating from *P. funiculosum* and *Aequorea victoria*, respectively, whereas *cel7b* and *bgl1* were of native *T. reesei* origin. Furthermore, *cel7b* was a cDNA version, whereas *bgl1* was a genomic DNA sequence that contained three introns. We thus introduced all possible gene types (i.e., cDNA, genomic DNA, native gene, and heterologous gene) to validate the robustness of expression of this multi-cistronic protein expression approach.

### Screening of Transformants by Fluorescence Identifies Putative Protein-Expressing Candidates

**Fig. 3.**
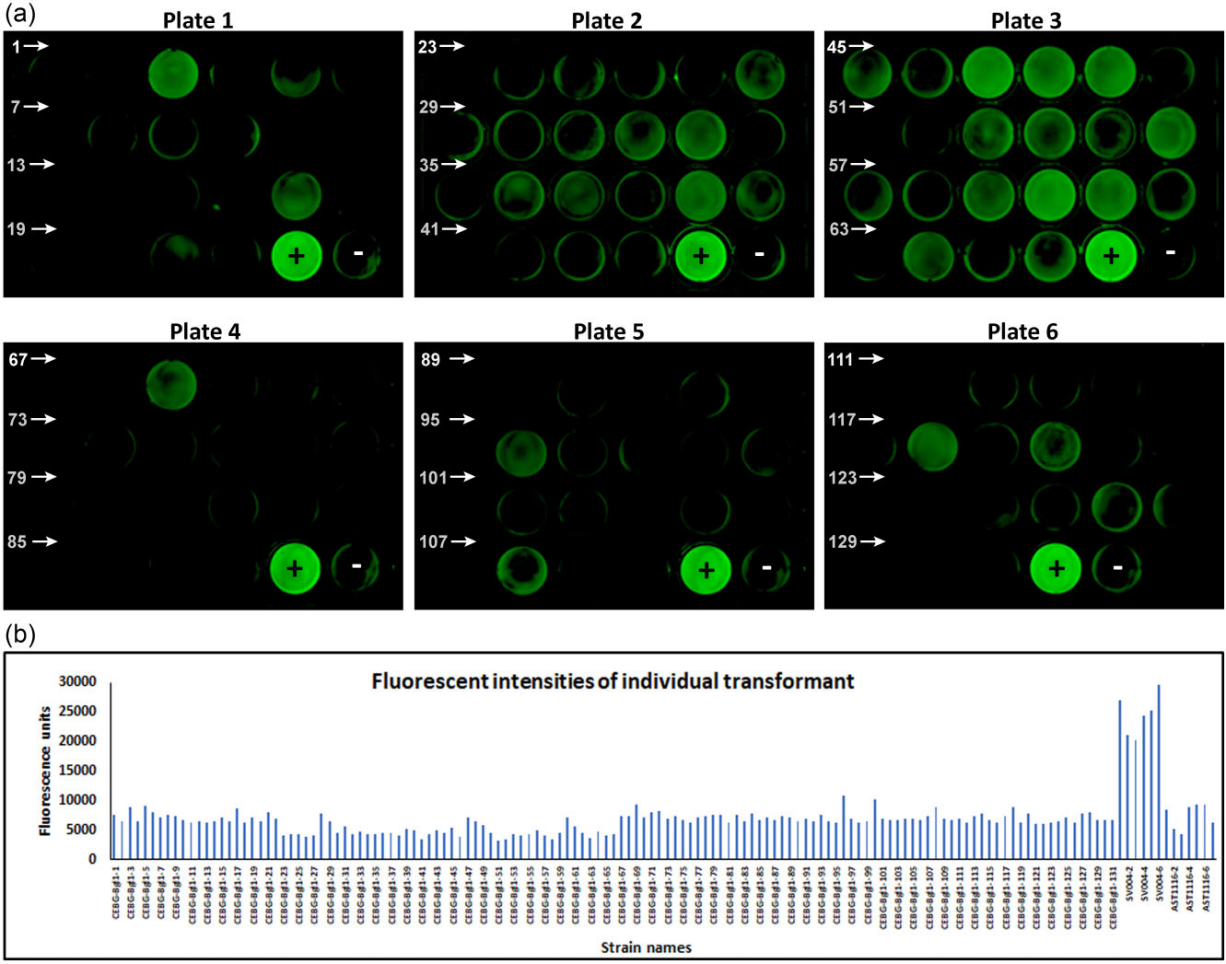
Fluorescence-based preliminary screening of pTrEno-CEBG transformants. **a.** 24-well plates containing MAG + H media were inoculated with spores and incubated for three days at 30°C. Fluorescence measurement was carried out using the FluorChem Q imaging station. Each plate contained a positive and a negative control. Numbers on each 24-well plate indicate transformant numbers. Only the first transformant on each row is labeled. +, CEL7A-2A-eGFP expressing strain SV004; –, AST1116 strain that does not express eGFP. **b.** Fluorescent intensities as quantified using the FLUOstar Omega plate reader. Alternate bars labeled on the X-axis represent individual transformants.

Considering that the four-gene containing plasmid, pTrEno-CEBG, was a very large construct (∼14.5 kb), we anticipated that a non-standard screening approach would be required to identify transformants that would express all four genes at the same time, due to the possibility of fragmented recombination occurring during the transformation of DNA into the cell. A preliminary screening was performed to determine the fluorescence of these transformant mycelia. As the 2A-peptide translation is contiguous and sequential, the expression of the terminal eGFP is a good indication of the translation of the upstream genes. We obtained 131 transformants based on selection on PDHX media plates. Each of these transformants was grown in 24-well plates containing MAG + H liquid media to allow mycelial growth formation. Fluorescence intensities of these colonies were measured using a FluorChem Q imaging station and a FLUOstar Omega plate reader. We observed several colonies that showed higher fluorescence than the control strain AST1116 using the FluorChem Q imaging instrument. Specifically, 25 transformant mycelia showed visibly higher fluorescence (Fig. 3a) than AST1116. However, based on fluorescence measurements using the FLUOstar Omega plate reader, only two transformants (#96 and #100, Fig. 3b) showed substantially higher fluorescence (measured in fluorescence units, F.U.) than the AST1116 strain, suggesting poor correlation between the two instrumentation approaches. The data also suggested that the overall levels of eGFP expression were low in these transformant colonies, and explicit identification of CEBG-expressing colonies could not be made at this point. Therefore, secondary screening and confirmation of a subset of the transformants were required to determine the true protein-expressing transformants.

### Secondary Fluorescence-Based Screening Identifies True Protein-Expressing Transformants

**Fig. 4.**
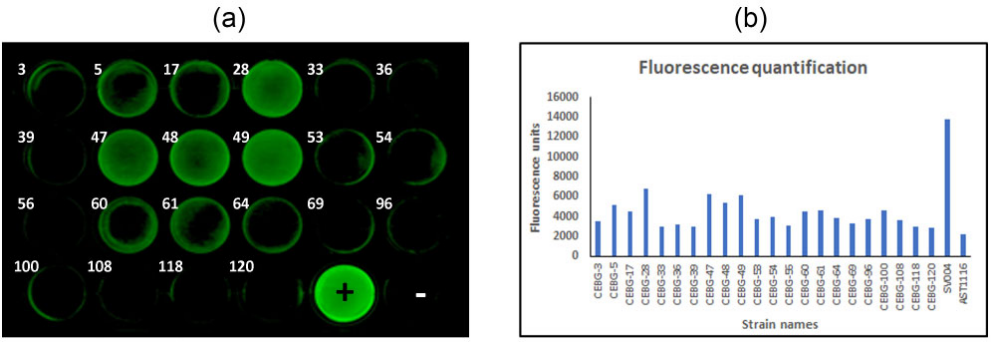
Secondary screening of down-selected transformants for CEBG-expression. **a.** 24-well plates containing MAG + H media were inoculated with spores and incubated for three days at 30°C. Fluorescence measurement was carried out using the FluorChem Q imaging station. Each plate contained a positive and a negative control. Numbers on the wells represent transformant numbers. +, CEL7A-2A-eGFP expressing strain SV004; –, AST1116 strain that does not express eGFP. **b.** Fluorescent intensities as quantified using the FLUOstar Omega plate reader. Bars represent individual transformants.

Based on their fluorescence intensities (Fig. 3) from the first round of screening, we selected 22 colonies that had marginally high fluorescence compared to AST1116 for further analysis. Mycelial fragments were transferred to fresh media in 24-well plates, along with the controls, SV004 and AST1116, and allowed to grow for three days at 30°C. Fluorescence measurements were carried out on these mycelia. Among this small batch of colonies, we observed that there was better differentiation between fluorescent and non-fluorescent mycelia (Fig. 4). Interestingly, the mycelial fluorescence of the transformants was quite consistent between the first and the second round of screening (Figs. 3 and 4), supporting the reproducibility of the two screening techniques. Four of the 22 transformants (#28, #47, #48, and #49) showed higher fluorescence than the negative control (AST1116) using both fluorescence measurement techniques (Fig.4a and b). A few other colonies #5, #17, #54, #60, #61, and #100 showed higher fluorescence than the AST1116 strain, although lower than the four transformants (#28, #47, #48, and #49) based on their fluorescence unit measurements (Fig. 4b). The rest of the colonies showed the lowest fluorescence in the range of AST1116 as measured by the two techniques. The fluorescence-based measurements identified a subset of transformants with a range of eGFP expression levels. Expression of eGFP could be enabled either by the *eGFP* gene by itself, in the case of fragmented integration, or by the complete four-gene-cassette in the case of full-cassette integration. To identify transformants that contained the full-gene cassette, we carried out genomic-PCR analysis for twelve transformant candidates. We observed that the colonies #28, #47, #48, #49, #61, #64, and #100 showed the expected PCR product of 6.5 kb. Colony #100 showed extremely faint PCR amplification (Supplementary Fig. 1). None of the other transformants showed the 6.5 kb product. These data clearly showed that the higher the fluorescence of mycelia, the higher the chances are of transformants containing the full-gene cassette. Nevertheless, the fluorescence of the full-gene cassette containing transformants, in general, was significantly lower than the fluorescence of the two-gene cassette (SV004 in Figs. 3 and 4), suggesting that eGFP expression and fluorescence are directly proportional to the length of the expression cassette. This could be attributed to the number of copies that the four-gene integrant (i.e., the CEBG transformants) versus the two-gene integrant (SV004) contain, a factor that cannot be controlled in a random integration approach like the one used in this study. However, it can be definitively concluded that fluorescence screening is more efficient when the gene cassette involves a lesser number of genes as opposed to multiple genes, as done in this study.

### Immunoblotting Reveals Expression of All the Three Cellulases

**Fig. 5.**
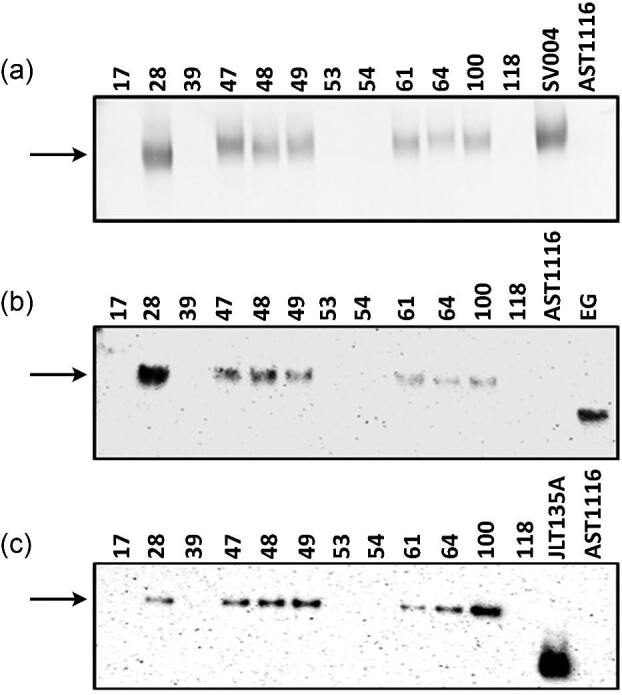
Immunoblot analysis for detecting protein expression in the selected CEBG transformants. **a.** CEL7A expression was determined by hybridizing with anti-CBH1 antibody raised in rabbit. Colorimetric detection was carried out using goat anti-rabbit AP-conjugated secondary antibody. **b.** EG1 expression was determined by hybridizing with anti-EG1 antibody raised in mouse. Chemiluminescence detection was carried out using goat anti-mouse HRP-conjugated secondary antibody. **c.** BGL1 expression was determined by hybridizing with an anti-His antibody raised in mouse. Chemiluminescence detection was carried out using goat anti-mouse HRP conjugated secondary antibody. Numbers represent individual transformants. SV004, CEL7A-2A-eGFP transformed strain; AST1116, *cel7a*-deleted *Trichoderma reesei* strain; JLT135A, his-tagged CBH1-expressing *T. reesei* strain; EG, purified EG1 protein.

Because all the cellulases that we expressed in the CEBG construct were extracellular enzymes, we carried out immunoblot analysis of the cell-free broths to determine the presence of each of the expressed proteins. For the detection of CBH1 and EG1, custom-raised anti-Cel7A and anti-Cel7B antibodies were used. We detected the presence of BGL1 using an anti-His antibody, as the corresponding protein contained a 6X-histidine tag cloned into the C-terminal terminus of the protein. eGFP protein was detected using an anti-eGFP antibody. We observed that colonies #28, #47, #48, and #49, which showed the highest fluorescence as well as the presence of the full-gene cassette (Fig. 4 and Supplementary Fig. 1) also showed detectable levels of CBH1, EG1, and BGL1 proteins in the immunoblot analyses (Fig.5a–c) at their expected molecular weights (∼53 kDa for CBH1, ∼46 kDa for EG1, and ∼76 kDa for BGL1). The levels of expression also correlated to the intensities of the bands on the immunoblots, except for BGL1 where #28 had slightly lower protein levels detected. Interestingly, even transformants #61, #64, and #100 showed detectable levels of all three cellulases, even though the level of fluorescence was lower than #28, #47, #48, and #49 (Fig. 4). The full-gene product was amplified for all three transformants (#61, #64, and #100) in the PCR analysis (Supplementary Fig. 1). The weaker amplification efficiencies (based on intensities of the PCR bands) in the genomic PCR analysis could be attributed to varied copy numbers combined with the length of the amplification product in these transformants. We therefore decided to evaluate if protein expression could be directly related to gene copy numbers. We carried out qPCR analysis of two genes, *cel7b* and *cel3a*, along with a single copy native gene *act1* on a subset of these transformants to determine the number of copies of the cassette that each of the transformants harbored in their genome. Based on our analysis, we determined that the copy numbers varied from 1 to 3 in the different transformants (Supplementary Fig. 2). Interestingly, transformants that showed detectable levels of proteins (#28, #47, #48, #61, #64, and #100, Fig. 5) showed 2–3 copies of the genes. An exception was transformant #49 that showed the presence of only one ectopic copy of the two genes. Those transformants showing <2 copies (#17, #39, #53, #54, and #118) showed no detectable protein expression. Transformants #39, #53, and #54 showed a single copy of the two ectopic genes *cel7b* and *cel3a*, even though they did not show the presence of the 6.5 kb PCR product (Supplementary Fig. 1), which explains the undetectable protein expression by these transformants. Fragmentation of the transformed CEBG construct could likely explain the detection of the additional *cel7a* and *cel3a* copies in the genome. These observations confirmed that protein expression can be directly attributed to the number of copies in this fungus. Additional transcriptional and translational controls could be exerted by factors such as promoter strength, location of integration, and codon usage.

These results showed that not only were all three of the cellulases expressed, they were also cleaved efficiently at the 2A-peptide sequences giving rise to intact proteins at their expected molecular weights and secreted efficiently in these transformants. This observation is especially important considering that the 2A-peptide-based protein expression results in the addition of residual 2A-peptide residues to the processed proteins that, in theory, could affect structure and/or function of the expressed proteins to different extents (Subramanian et al., 2017). Furthermore, our results also showed that the native secretion peptides are recognized in this system provided they are cloned downstream of the 2A-peptide sequences, which in theory will add a single proline residue to the N-terminal end of the expressed proteins (Donnelly et al., 2001c; Subramanian et al., 2017).

### Proteins Expressed from the 2A-Peptide Construct are Catalytically Active

**Fig. 6.**
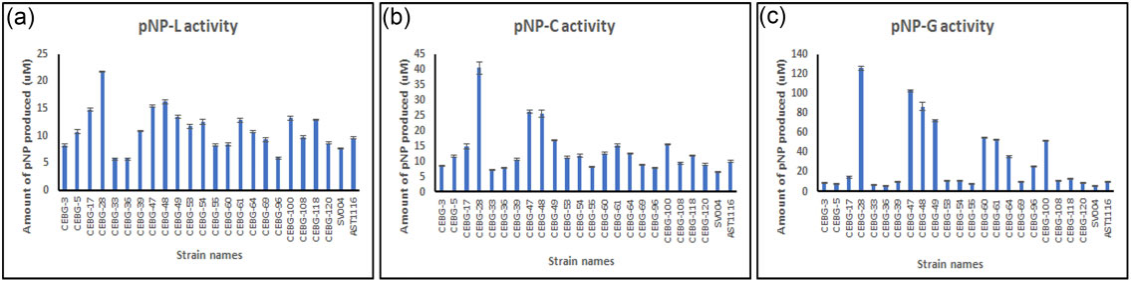
pNP activity assays of CEBG transformants. **a.** pNP-L activity. **b.** pNP-C activity. **c.** pNP-G activity. 25 µL of cell-free broth was used for the activity assays. Bars represent the micromoles of pNP released during the reaction as measured at 405 nm. SV004, CEL7A-2A-eGFP transformed strain; AST1116, *cel7a*-deleted *Trichoderma reesei* strain. Data represent analysis carried out in technical duplicates for each transformant.

To determine if the proteins expressed by the fluorescent and full-gene cassette-containing transformants were functional, we carried out activity assays using *p*NP-glycoside derivatives as semi-differentiating substrates. Specifically, *p*NP-L, *p*NP-C, and *p*NP-G were used as surrogate substrates for the determination of CBH1, EG1, and BGL1 activities, respectively. These assays are based on the ability of CBH1, EG1, and BGL1 to release the *p*-nitrophenol moiety from their respective aglycones, after which it is monitored by the development of a yellow color under alkaline conditions. While the combinations of each enzyme and substrate are not highly specific, enough differentiation can be made to be indicative of the individual activities. Activity analysis was carried out using 25 µL of the cell-free broth. We observed that transformants #28, #47, #48, and #49 showed higher activity levels on all three substrates (Fig. 6). The differences in activities between these transformants were more obvious when *p*NP-C and *p*NP-G were used as substrates; in contrast to the case where *p*NP-L was used as the substrate, where several colonies showed higher levels of apparent activity than the controls (Fig.6a–c). Hydrolysis of the *p*NP-G substrate showed the highest specificity among the three activity assays tested, with the highest fluorescing colonies also demonstrating the highest BGL activities (Figs. 4 and 6c). Nevertheless, there were other transformants, such as #60, #61, #64, and #100, which also showed higher apparent activity using *p*NP-C and *p*NP-G as substrates (Fig. 6b and c). Not surprisingly, these transformants also showed marginally higher fluorescence than the negative control, as well as detectable levels of the three proteins by western blotting (Fig. 5). These results clearly showed a correlation between fluorescence measurements and functional activity of the expressed proteins, suggesting that fluorescence measurements could be reliably used as a tool for monitoring gene expression of even longer gene cassettes (at least up to four genes) in this system.

Cellulase activity is typically exhibited by the concerted action of exoglucanases (CBH1, E.C. 3.2.1.1) that catalyze the processive end-wise hydrolysis of 1,4-β-glucosidic bonds in cellulose, endo-glucanases (endo-β-1,4-glucanase, E.C. 3.2.1.91), which catalyze the hydrolysis of internal glucosidic bonds in cellulose, and finally the β-glucosidases (β-D-glucoside glucohydrolase, E.C. 3.2.1.21) that hydrolyze the liberated cellobiose into free glucose molecules. AZCL-HE-cellulose is a chromogenic insoluble form of cellulose that is typically used for the detection of EG activity (Inokuma et al., 2014; Quay et al., 2011). Upon depolymerization and solubilization by endocellulases, the blue dye is released into the media. We used this substrate to evaluate the activity of EG1 on a more close-to-real-world substrate to determine if its function is affected by the “intra-2A-peptide” location within the gene cassette (i.e., second gene in the 2A-peptide cassette).

**Fig. 7.**
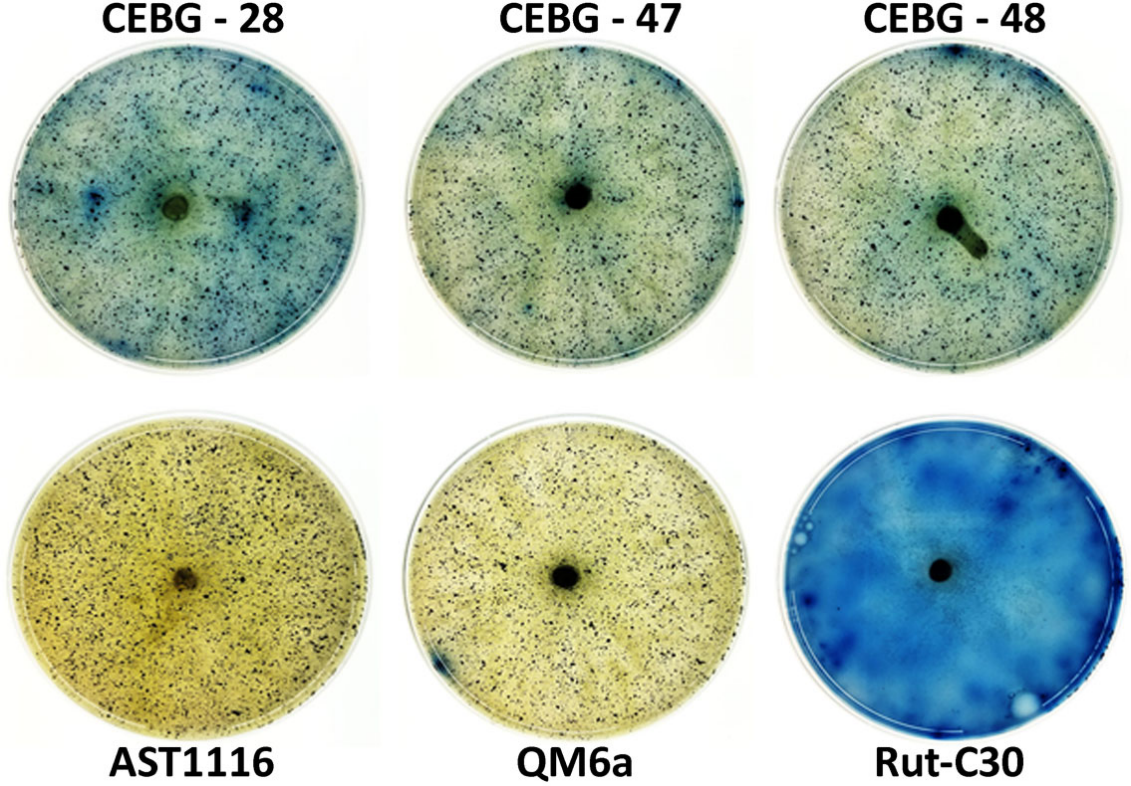
Whole-cell AZCL-HE-cellulose activity assay. Three CEBG transformants #28, #47, and #48 were tested for AZCL-HE-cellulose breakdown on agar plates by placing a mycelial plug in the center of media plates containing the chromogenic substrate. After incubating the plates for six days at 30°C, plates were visualized for blue coloration. Higher levels of blue coloration indicate higher endo-glucanase activities. AST1116, *cel7a*-deleted *Trichoderma reesei* strain; QM6a, wild-type *T. reesei* strain; Rut-C30, cellulase hyper-producing strain of *T. reesei*.

Fungal mycelia were transferred from PD plates to AZCL-HE-cellulose containing MA media and allowed to grow for six days to observe growth as well as the development of blue color. We observed that all three CEBG transformants grew on this substrate, while also showing blue coloration due to the breakdown of the chromogenic substrate (Fig. 7). However, neither the parent strain QM6a nor the *cel7a*-deleted strain, AST1116, showed blue coloration on these plates during the same time period, suggesting that the levels of the native cellulase system are insufficient to breakdown AZCL-HE-cellulose to detectable levels. This is likely a result of legacy repression of the control strains’ native cellulase systems resulting from extended growth on PDA-based media. This also indicated that activity in the heterologous strains was due to the constitutively expressed heterologous cellulases and not from induction of the native cellulases. Among the three transformants tested, the CEBG strain #28 showed the highest coloration on AZCL-HE-cellulose plates, which coincided with its highest fluorescence signal (Fig. 4) and higher protein levels (Fig. 5). Strains #47 and #48 showed lower levels of blue coloration, coinciding with lower pNP-C activity data (Fig. 6b). It is difficult to speculate if breakdown of AZCL-HE-cellulose is solely due to the EG activity or is also augmented by the expression of CBH1 and the BGL1 enzymes, although overexpression of CBH1 alone in the AST1116 background strain (i.e., JLT102A strain) in glucose grown conditions does not lead to breakdown of this substrate (Supplementary Fig. 3). Nevertheless, this observation clearly suggests that the function of EG1 is not affected by the internal positioning of the corresponding gene in the 2A-peptide cassette. This is particularly important because protein function and/or stability may be affected by the N- or C-terminal location within the 2A-peptide cassette (Subramanian et al., 2017). We also tested the activity of the cellulase hyperproducing strain Rut-C30, which produces high levels of these enzymes (i.e., even under glucose-grown conditions). We observed that in comparison to the CEBG transformants, Rut-C30 showed several-fold higher blue coloration, indicating overall higher levels of enzymes, including the EG(s) (Peterson & Nevalainen, 2012).

### Protein Expression Levels Vary Between the Three Cellulases When Expressed from a 2A-Peptide Cassette

**Fig. 8.**
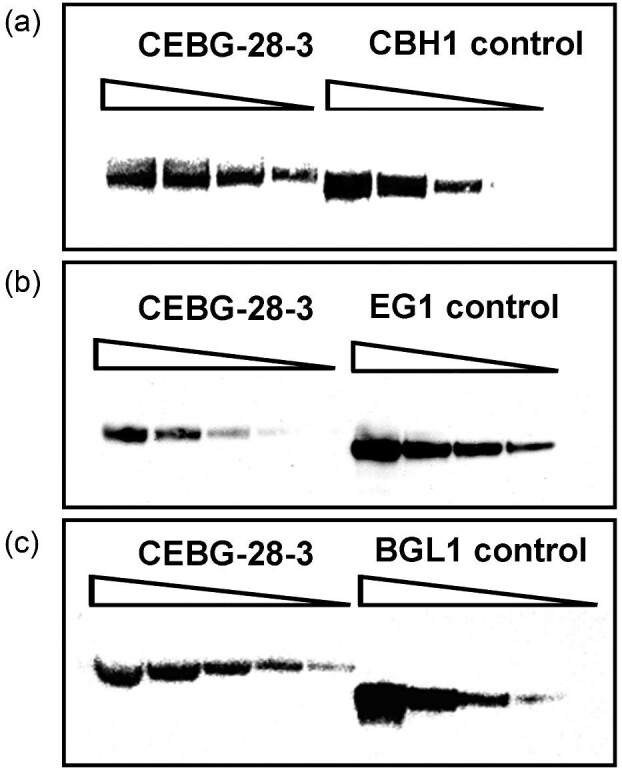
Quantification of cellulase expression by immunoblotting. Serial dilutions of CEBG #28 cell-free broth were separated on SDS-PAGE gel in parallel with dilutions of known concentrations of control proteins followed by immunoblotting. Bands were quantified using FluorChem Q imaging station analysis software. Unknown concentrations of the three proteins CBH1, EG1, and BGL1 were determined using a standard curve generated with the control proteins run on the same gel. **a.** Determination of CBH1 concentration in the cell-free broth. Protein amounts of 25, 12.5, 6.25, and 3.125 µg of cell-free broth and 0.6, 0.3, 0.15, and 0.075 µg of purified CBH1 proteins were used. Western blotting was done using anti-CBH1 antibody. **b.** Determination of EG1 concentration in the cell-free broth. Protein amounts of 25, 12.5, 6.25, 3.125, and 1.56 µg of cell-free broth and 1.2, 0.6, 0.3, and 0.15 µg of purified EG1 proteins were used. Western blotting was done using an anti-EG1 antibody. **c.** Determination of BGL1 concentration in the cell-free broth. Protein amounts of 100, 50, 25, 12.5, and 6.5 µg of cell-free broth and 1.2, 0.6, 0.3, and 0.15 µg of 6X histidine-tagged CBH1 proteins were used. Western blotting was done using an anti-His antibody.

**Fig. 9.**
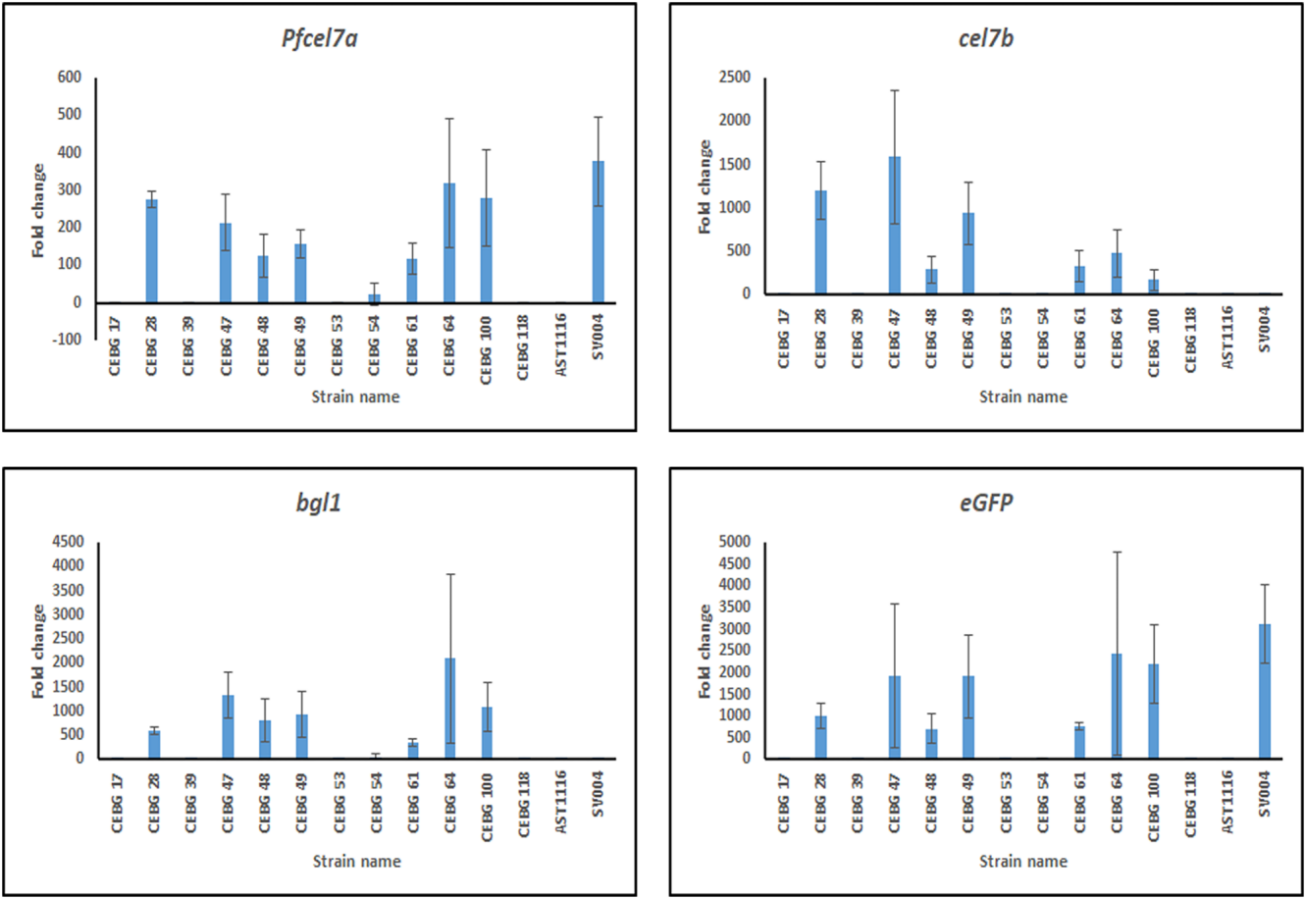
Reverse transcription quantitative PCR analysis. RT-qPCR analysis was carried out for the four genes *Pfcel7a, cel7b, bgl1*/*cel3a*, and *eGFP* using gene-specific primers (Supplementary Table 1). Whereas the *cel7a* sequence originated from *Penicillium funiculosum, cel7b*, and *bgl1* sequences were from *Trichoderma reesei*. eGFP sequence originated from *Aequorea victoria*. Ct values were normalized against the actin gene. Fold changes were calculated using the ΔΔct approach. SV004, CEL7A-2A-eGFP transformed strain; AST1116, *cel7a*-deleted *T. reesei* strain.

To determine the levels of CBH1, EG1, and BGL1 proteins expressed within the 2A-peptide construct, we carried out large-scale growth (8-L) of CEBG strain #28 to obtain higher amounts of protein. Cell-free broth was concentrated to 200 mL total volume, followed by protein content measurement using the BCA analysis protocol. A series of dilutions of strain #28 cell-free broth were separated on SDS-PAGE gels in parallel with known concentrations of control proteins representing each of the enzyme class. Based on immunoblotting, we determined the abundances of each of CBH1, EG1, and BGL1 to be 6.72, 0.60, and 0.38 µg, respectively, per 100 µg of concentrated total cell-free broth (Fig. 8). These titers correspond to 128 pmoles, 13 pmoles, and 5 pmoles of each protein, respectively. The ratio of the expressed proteins turned out to be ∼18:2:1, which suggested that the protein expression levels drop almost 20-fold from protein 1 (i.e., CBH1) to protein 3 (i.e., BGL1) within the 2A-peptide construct. Remarkably, eGFP, which is the fourth protein in the series, can still be detected by fluorescence even at <18-fold of protein 1. It is generally considered that stoichiometrically similar protein levels should be obtained using the 2A-peptide approach because all four proteins arise from a single mRNA transcript. To eliminate the possibility that the different protein levels observed could be a factor of different transcriptional levels, we carried out real-time RT-qPCR analysis on each of the associated genes within the gene cassette. We observed that detectable levels of transcripts, in comparison to the wild-type control strain QM6a, were only observed for transformants #28, #47, #48, #49, #61, #64, and #100 (Fig. 9). CEBG #54 showed detectable and higher-than-background levels of transcripts. The real-time RT-qPCR analysis data coincided well with those transformants that also showed detectable levels of proteins, as well as the significant cellulase activity observed in those respective transformants (Figs. 5 and 6). For the most part, it appeared that the transformants with the highest copy numbers also showed higher transcript levels (Fig. 9). As expected, SV004, which harbored only the *Pfcel7a* and the *eGFP* genes, showed only detectable levels of the respective gene transcripts; no detectable transcription of *cel7b* and *cel3a* was observed in this strain due to the fact that these transformants were grown in glucose conditions, where the entire cellulase machinery is suppressed, except for the heterologous genes *PfCel7a* and *eGFP* that are under the control of the constitutive *eno1* promoter. This suggested a close correlation between gene copy, transcription, and protein activity observed in the transformants. However, different absolute protein amounts have been observed in this study for the four different proteins, which is very intriguing. This result suggests a possible loss of translational efficiency when encountering long gene transcripts, similar to that observed in mouse cell lines (Liu et al., 2017). This result could be attributed to translational controls exerted by codon usage and/or protein folding and/or stability issues. Nonetheless, engineering ribosomal binding sites into bicistrons have shown to improve expression of individual genes in bacterial operon systems (Farasat et al., 2014; Jang et al., 2018; Shi et al., 2018). Internal ribosomal entry sites (IRESs) have also been identified in viral systems and have been implemented in eukaryotic expression vectors for two-protein expression systems (Al-Allaf et al., 2019; Attal et al., 1999; Bochkov & Palmenberg, 2006). Introducing IRES into the CEBG construct could enhance the translation of the downstream genes, although this would result in an increase in the size of the construct by >2.5 kb, considering that the IRES sequences are typically ∼600 bp long. This increase in plasmid/cassette size could affect the frequency of full-cassette integrations in the transformants. However, it would be worth testing the impact of IRES on the expression of such multi-gene cassettes for fungal protein expression. It should be noted that AST1116 is a QM6a derivative, which is a glucose-repressed *T. reesei* strain. Moreover, this strain is not considered to be an industry standard for cellulase expression. We have used AST1116 as our model host strain because it lacks CEL7A, thereby aiding in the detection of ectopically expressed CEL7A, such as the one expressed in this work. Expression of the four-gene construct in hyper-cellulase expression strains, such as Rut-C30, should result in several-fold higher expression levels compared to QM6a, but differentiation from background native cellulase activity would be extremely difficult. However, this work has provided a proof-of-concept for the application of this approach toward future multi-gene pathway expression in this fungus.

## Conclusion

We have shown that multiple proteins can be expressed simultaneously in *T. reesei* using the 2A-peptide approach. In this work, we have developed a plug-and-play expression vector, capable of expressing up to five proteins under a common promoter and a terminator for the industrial work horse fungus, *T. reesei*. We have particularly exploited this technique to express three enzymes critical for cellulose degradation, which include a CBH1, a EG1, and a BGL1. We have clearly shown that protein expression and activity are directly related to the number of gene copies harbored in these transformants, which also correlates with the observed transcript levels of the respective genes. However, protein levels appear to drop off for the individual proteins within the cassette possibly due to the translational and post-translational constraints caused by the overall length of the gene cassette and/or other translation and/or protein stability issues. Overall, the shorter the expression cassette, the higher the translational level of the individual genes, and vice versa is a logical conclusion that could be derived from this work. This conclusion is further supported by the fact that the SV004 strain, which expresses only two genes, shows the highest levels of fluorescence, transcript, and protein levels. We have also demonstrated that eGFP can be used reliably to monitor expression of all proteins within the four-gene cassette, as observed by the detection of several transformants showing detectable expression of all four proteins. Not only have these proteins been shown to be expressed and secreted, but we have also validated this approach by demonstrating the functionality of each of these proteins using enzyme assays. This work has clearly demonstrated that cellulase enzymes can be efficiently expressed, processed, and secreted in *T. reesei* despite containing additional amino acids introduced during the “ribosomal-skip” mechanism occurring during the 2A-peptide translation event. For the first time, we have also been able to demonstrate the diversity of expression levels of each protein within the 2A-peptide construct in a fungal expression host. This approach is particularly applicable to those enzyme mixtures that require differential levels of protein and/or their activity rather than equimolar protein levels, such as the classical fungal cellulase system. Development of this protein expression tool would not only prove valuable for expressing multi-enzyme cocktails but also for expressing bioproduct synthesis pathways in *T. reesei*. Future work will involve improving expression levels of the enzymes within the 2A-peptide construct as well as testing expression levels in different *T. reesei* strains.

## Supplementary Material

kuac027_Supplemental_Files
