# Supplementary material for: A multi-plex protein expression system for production of complex enzyme formulations in Trichoderma reesei
Source: J Ind Microbiol Biotechnol. 2022 Dec 13;49(6):kuac027. doi: 10.1093/jimb/kuac027 (PMC9923369; doi:10.1093/jimb/kuac027)
Supplement: kuac027_Supplemental_Files [file kuac027_supplemental_files.zip › Supplementary Figure S1.docx]

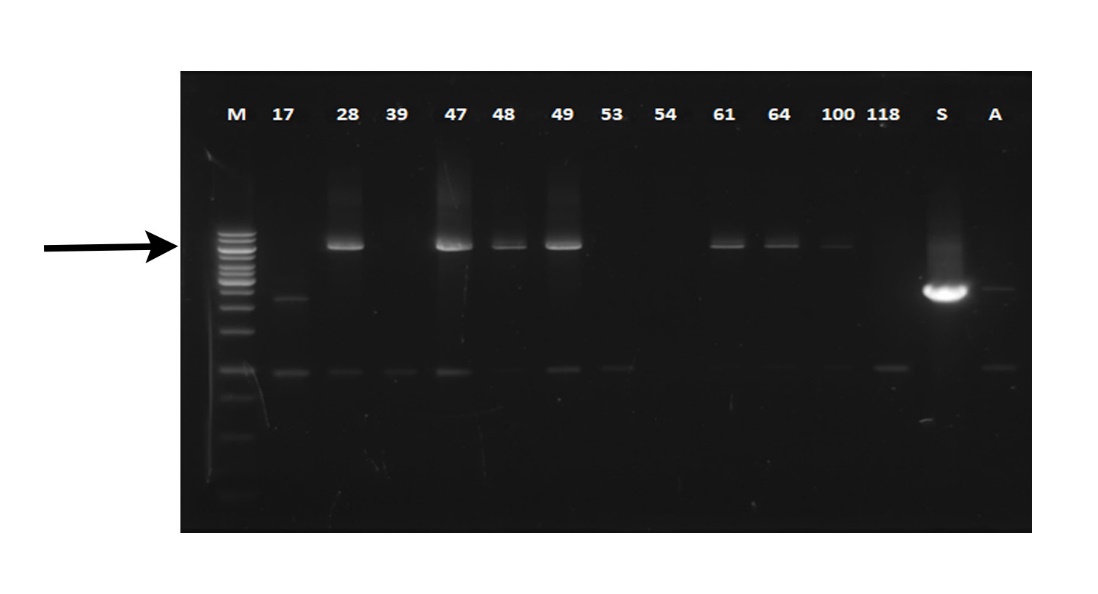


**Supplementary Figure S1. PCR analysis of transformants.**  PCR analysis was carried out on genomic DNA using flanking primers SV-82 and JL-383 to amplify the complete gene cassette. The expected product size is 6500 bp, which is indicated by the black arrow. Lanes marked by #s 28, 47, 48, 49, 61, 64 and 100 show the expected PCR product. Numbers above the lanes indicate transformant numbers. M, GeneRuler 1 kb DNA ladder; S, SV004 strain (AST1116 containing Cel7A-2A-eGFP); A, AST1116 (Cel7A deleted strain of QM6A).
