# Supplementary material for: A multi-plex protein expression system for production of complex enzyme formulations in Trichoderma reesei
Source: J Ind Microbiol Biotechnol. 2022 Dec 13;49(6):kuac027. doi: 10.1093/jimb/kuac027 (PMC9923369; doi:10.1093/jimb/kuac027)
Supplement: kuac027_Supplemental_Files [file kuac027_supplemental_files.zip › Supplementary Figure S2.docx]

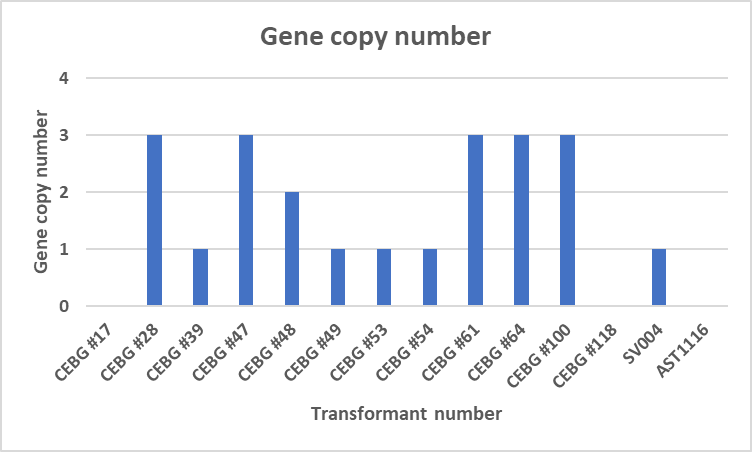


N

N

N

N

N

N

Y

Y

Y

Y

Y

Y

Y

Y

**Supplementary Figure S2. Gene copy number analysis.** Genomic DNA from individual transformants was used to amplify *cel7b*, *cel3a*, and *act1* genes using gene specific primers. Gene copies were normalized against the single copy gene *act1*. “Y” and “N” represent the presence and absence, respectively, of full-length CEBG PCR products (see Supplementary Figure S1) in the transformants. SV004, CEL7A-2A-eGFP transformed strain; AST1116, *cel7a*-deleted *T. reesei* strain.
