# Supplementary material for: A multi-plex protein expression system for production of complex enzyme formulations in Trichoderma reesei
Source: J Ind Microbiol Biotechnol. 2022 Dec 13;49(6):kuac027. doi: 10.1093/jimb/kuac027 (PMC9923369; doi:10.1093/jimb/kuac027)
Supplement: kuac027_Supplemental_Files [file kuac027_supplemental_files.zip › Supplementary Figure S3.docx]

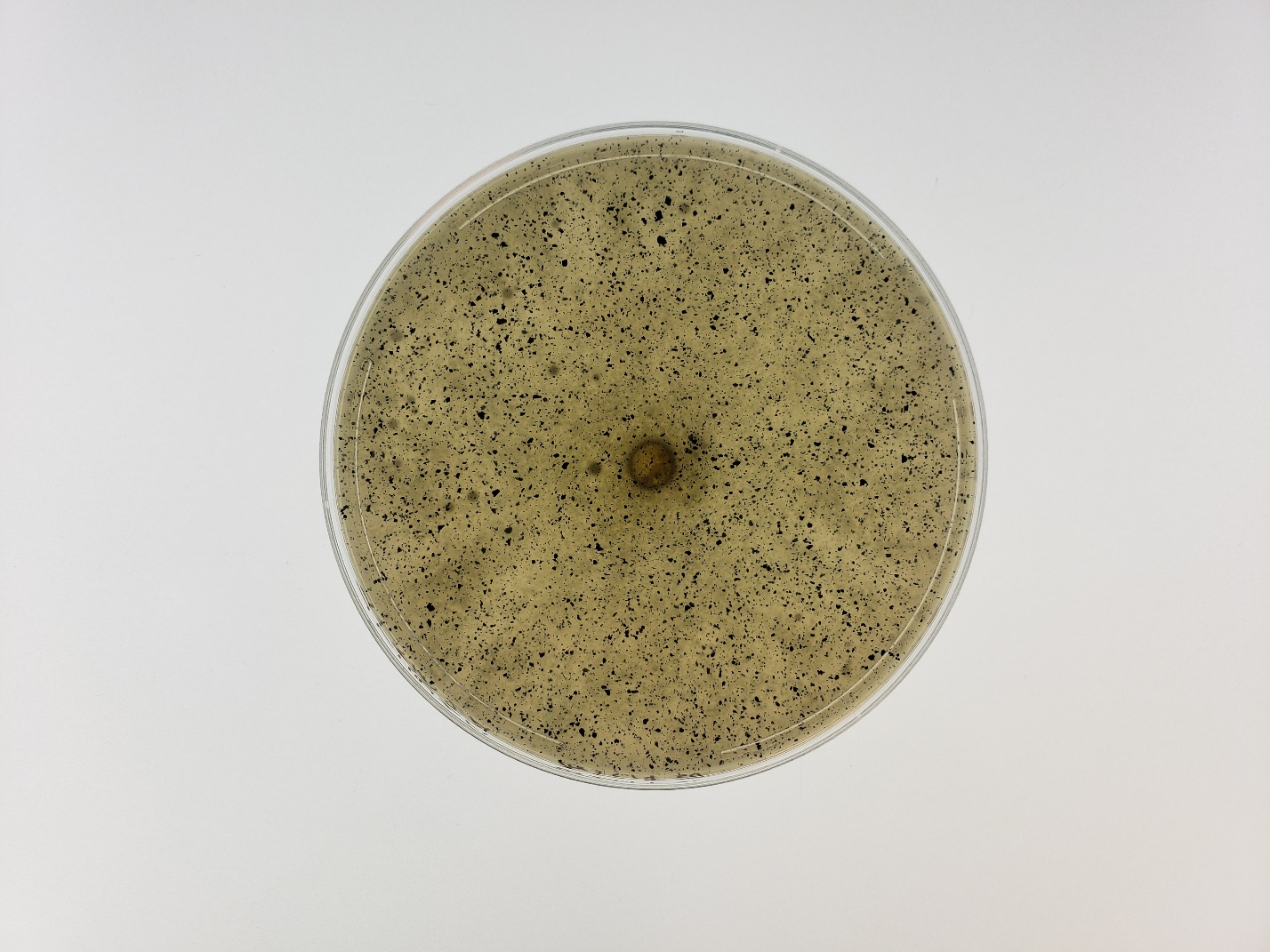


**Supplementary Figure S3. Whole-cell AZCL-HE-Cellulose activity assay using the CEL7A-expressing *T. reesei* strain JLT102A.** A mycelial plug of JLT102A strain was placed on AZCL-HE-Cellulose containing agar plates. After incubating the plates for 6 d at 30 ^o^C, plates were visualized for blue coloration.
