# Supplementary material for: A multi-plex protein expression system for production of complex enzyme formulations in Trichoderma reesei
Source: J Ind Microbiol Biotechnol. 2022 Dec 13;49(6):kuac027. doi: 10.1093/jimb/kuac027 (PMC9923369; doi:10.1093/jimb/kuac027)
Supplement: kuac027_Supplemental_Files [file kuac027_supplemental_files.zip › Supplementary Table S1.docx]

**Supplementary Table S1.** **Primers used in this work**

| **Primer name** | **Primer sequence (5’ – 3’)** | **Purpose** |
| --- | --- | --- |
| SV-33 | CGG AAT TAA TTA AAT GTC TGC CTT G | Plasmid cloning |
| SV-168 | CGA ATT CCA AAC ATT GAG AGT AGT AAG GG | Plasmid cloning |
| SV-230 | CGG GAT CCA TGG CGC CCT CAG TTA | Plasmid cloning |
| SV-231 | ACG CTA GCA AGG CAT TGC GAG TAG TAG | Plasmid cloning |
| SV-346 | ACC GGC ATG CAT GCG TTA CCG AAC AGC AG | Plasmid cloning |
| SV-347 | ACA CTA GTA TGG TGG TGG TGA TGG TGC GCT ACC GAC AGA GTG CTC GTC | Plasmid cloning |
| SV-246 | CGC GAT CGA TGG TCT CCA AGG G | Plasmid cloning |
| SV-79 | GCC TCT AGA TTA CTA CTT GTA CAG C | Plasmid cloning |
| SV-75 | GTA GTA ATC TAG AGG CTT TCG TGA C | Plasmid cloning |
| SV-278 | CCT TGG AGA CCA TCG ATC GCG GGC CAG GAT TGC TTT CGA CAT C | Plasmid cloning |
| SV-82 | GCT CCT CTG GCT GGA TTT TG | For PCR analysis of transformants |
| JL-383 | GAC CTG CGA CAG ACA ACC AA | For PCR analysis of transformants |
| SV-481* | CGG CAA CAT TGT CAT GTC TG | *T. reesei act1* |
| SV-482* | ACC GCT CTC GTC GTA CTC | *T. reesei act1* |
| SV-483* | GCG CTG CTG AAA TTT CCA C | *Pfcel7a* |
| SV-484* | TGA CAT AGG AGC TGC CGG | *Pfcel7a* |
| SV-492* | GCA CGT CGT CTT CTC CAA C | *T. reesei cel7b* |
| SV-493* | GGC ATT GCG AGT AGT AGT CG | *T. reesei cel7b* |
| SV-494* ^φ^ | CAA TGC GCT CGT CGA CGT | *T. reesei bgl1*/*cel3a* |
| SV-496* | GTT GAA CTT GGT GTA AGA CAG TCC | *T. reesei bgl1*/*cel3a* |
| SV-179* ^φ^ | CAT CAA GGT CAA CTT CAA GAT CC | *eGFP* |
| SV-208* ^φ^ | CTT GTA CAG CTC GTC CAT GC | *eGFP* |
| SV-479^φ^ | TCG TCC GTG ACA TCA AGG | *T. reesei act1* |
| SV-480 ^φ^ | TCC TTT CGG ACG TCG ACG | *T. reesei act1* |
| SV-490 ^φ^ | CAC CCT CAA CAC TAG CCA C | *T. reesei cel7b* |
| SV-491 ^φ^ | GTC TTG GAG GTG TCA ACG G | *T. reesei cel7b* |
| SV-495 ^φ^ | CCA CAG TCA TCC GTC AGT C | *T. reesei bgl1*/*cel3a* |

* represent primers used for real-time reverse transcription quantitative PCR analysis

^φ^ represent primers used for gene copy analysis.
